# A Merited Honor

**Published:** 1894-05

**Authors:** 


A MERITED HONOR.—We note, with pleasure, the honor bestowed upon Prof. Robt. Field LeMond, of Gross Medical College, Denver, Colorado, by his *alma mater*, the Hospital College of Medicine, Milwaukee, Wisconsin. Prof. LeMond has been invited to deliver the address to the Graduating class at the approaching commencement, June 19, '94.

Professor LeMond, several years ago, quietly slipped into the "Capital City," and when he left, it was noticed that one of Austin's fairest and most charming ladies was missing. He had linked his fate with that of Mrs. Mary E. Slaughter. This is why, in common with the many other friends of the fair partner of his life, we feel such a deep, sympathetic interest in his elevation to the pinnacle of fame. May God speed the doctor, is our sincere wish. F. S. C.

